# Acupuncture of different treatment frequency in knee osteoarthritis: a protocol for a pilot randomized clinical trial

**DOI:** 10.1186/s13063-019-3528-8

**Published:** 2019-07-11

**Authors:** Lu-Lu Lin, Jian-Feng Tu, Jia-Kai Shao, Xuan Zou, Tian-Qi Wang, Li-Qiong Wang, Jing-Wen Yang, Ning Sun, Cun-Zhi Liu

**Affiliations:** 1 School of Acupuncture-Moxibustion and Tuina, Beijing University of Chinese Medicine, 11 Beisanhuan East Road, Chaoyang District, Beijing, 100029 China; 2 Department of Acupuncture and Moxibustion, Beijing Hospital of Traditional Chinese Medicine affiliated to Capital Medical University, Beijing, China

**Keywords:** Knee osteoarthritis, Acupuncture, Treatment frequency, Clinical trial

## Abstract

**Background:**

This study aims to determine whether 3 sessions per week of acupuncture treatment is superior to 1 session per week of acupuncture treatment for symptomatic outcomes in knee osteoarthritis.

**Methods/design:**

This is a two parallel-group, assessor-blinded, randomized controlled trial. Sixty patients with knee osteoarthritis (Kellgren–Lawrence grade II or III) will be recruited and randomly allocated to receive 24 or 8 sessions (group M or group L) of acupuncture treatment in a 1:1 ratio. Patients in group M will receive 3 sessions per week of acupuncture for 8 weeks. Patients in group L will receive acupuncture once per week for 8 weeks. The primary outcome is the response rate—the percentage of patients achieving a decrease ≥ 2 points on a numerical rating pain scale and a decrease ≥ 6 points in the Western Ontario and McMaster Universities Osteoarthritis Index function score at 8 weeks compared with baseline. Secondary outcomes include pain, function, overall effect, quality of life, and treatment credibility and expectancy.

**Discussion:**

Three sessions per week of acupuncture treatment may be superior to 1 session per week of acupuncture treatment for symptomatic outcomes in knee osteoarthritis. Results of the study will be of great importance for the guidelines of clinical therapy.

**Trial registration:**

Clinicaltrials.gov, NCT03359603. Registered on 1 December 2017.

**Electronic supplementary material:**

The online version of this article (10.1186/s13063-019-3528-8) contains supplementary material, which is available to authorized users.

## Background

Knee osteoarthritis is a common and disabling condition, particularly in people over 50 years of age [1]. The estimated lifetime risk for knee osteoarthritis is approximately 40% in men and 47% in women [2]. In addition to the personal burden of knee osteoarthritis, there are substantial direct and indirect health-care costs particularly in terms of employment status, productivity and joint replacement surgery, making knee osteoarthritis a substantial public health problem [3].

There is currently no consensus on the best treatment to improve knee osteoarthritis symptoms. Pharmacologic modalities used to treat the symptoms of this disorder are associated with various side effects such as renal toxicity, serious gastrointestinal complications and cardiovascular events [4]. In fact, patients generally do not like taking medicines [5]. People with knee osteoarthritis prefer nonpharmacological options for pain relief and usually choose complementary medicine [6].

Nonpharmacological methods such as education and self-management, exercise and physical activity are recommended in the guidelines [7, 8]. However, the effect of exercise treatment decreases over time [9] and self-management education programs result in no or small benefits in people with knee osteoarthritis [10]. In addition, these methods as complex interventions can decrease adherence for individuals [11].

Acupuncture is one of the most common complementary and alternative medical therapies and a popular treatment for pain and dysfunction associated with musculoskeletal conditions, and the effects of acupuncture are maintained over time [12, 13]. In a systematic review of osteoarthritis guidelines, five of the eight guidelines that considered acupuncture recommended it as an osteoarthritis treatment modality [4]. However, the benefits of acupuncture remain highly controversial. In a meta-analysis of acupuncture for knee osteoarthritis including 10 randomized controlled trials with a total of 2007 patients from 1994 to 2014, the acupuncture group had significantly improved overall pain and physical function scores as compared with the sham acupuncture group [14]. In contrast, another meta-analysis and several large trials indicated that real acupuncture had no significant effect on osteoarthritis pain and function as compared to sham acupuncture [15–17].

Based upon previous high-quality studies, we speculate that the clinical effectiveness of acupuncture for knee osteoarthritis may relate to treatment frequency. The treatment frequency in acupuncture trials with knee osteoarthritis which showed negative results [17, 18] is significantly lower than that in other trials showing positive results [19, 20]. Furthermore, acupuncture is superior to sham acupuncture on symptomatic outcomes in patients with chronic pain who received 3 or more sessions of treatment per week [21–25]. Meanwhile, 1 session or no acupuncture session per week usually showed no significant improvement in pain with acupuncture compared with sham acupuncture [17, 18]. However, there are no currently universally accepted treatment frequency criteria. The frequency of acupuncture treatment in patients with knee osteoarthritis is no more than twice per week in most trials, while it is usually 3–5 sessions per week in clinical practice in China. Studies also suggested that acupuncture treatments once a day or every other day maybe reasonable in chronic painful conditions [26]. Accordingly, we designed the current trial to compare the effects of different acupuncture sessions (3 sessions per week versus 1 session per week) in a pilot randomized controlled trial of knee osteoarthritis. The hypothesis is that 3 sessions per week compared with 1 session per week of acupuncture treatment will lead to better symptomatic outcomes.

## Methods

### Study design

**Fig. 1:**
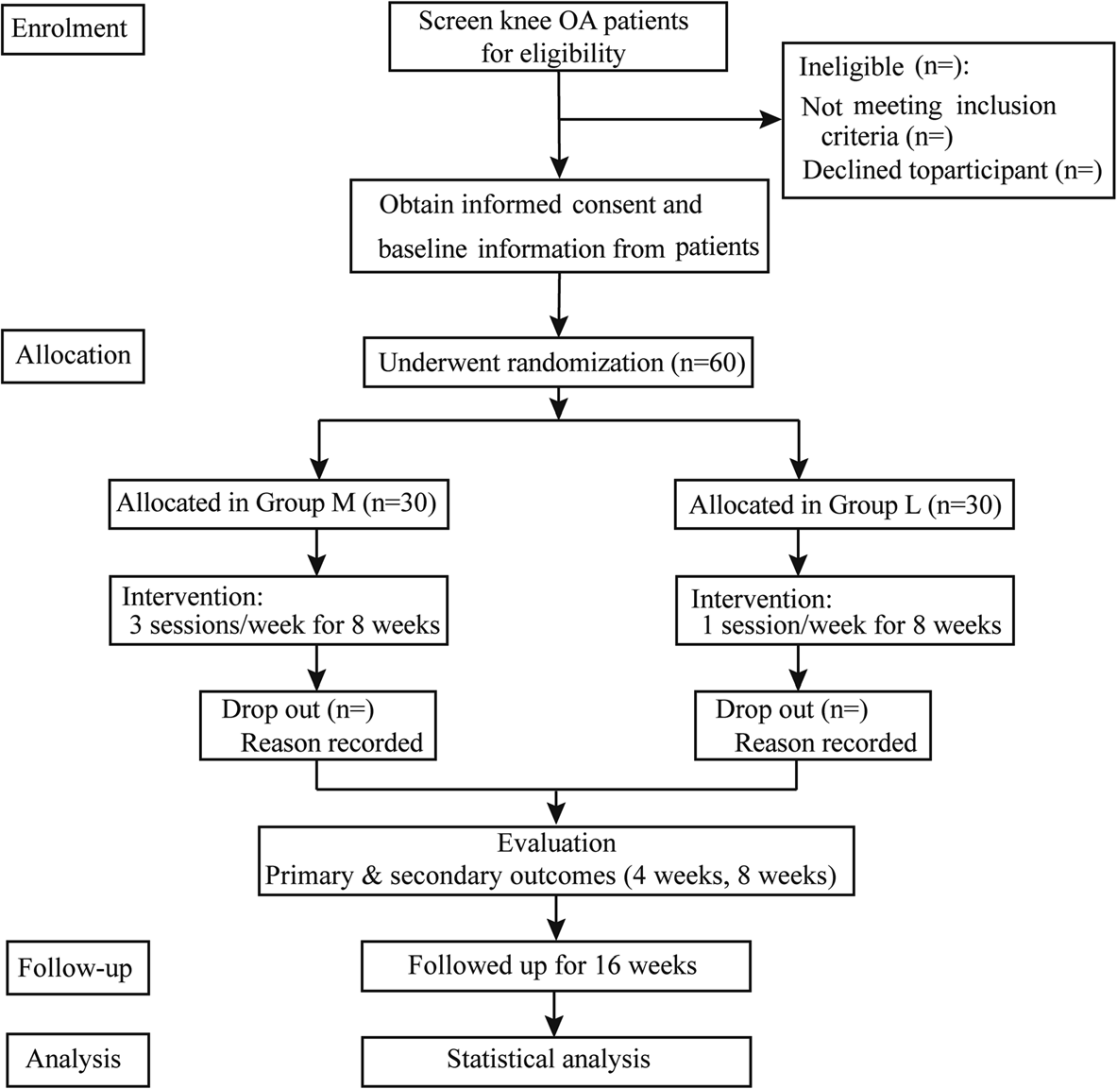
Trial flow chart: Consolidated Standards of Reporting Trials CONSORT diagram. OA osteoarthritis

**Fig. 2:**
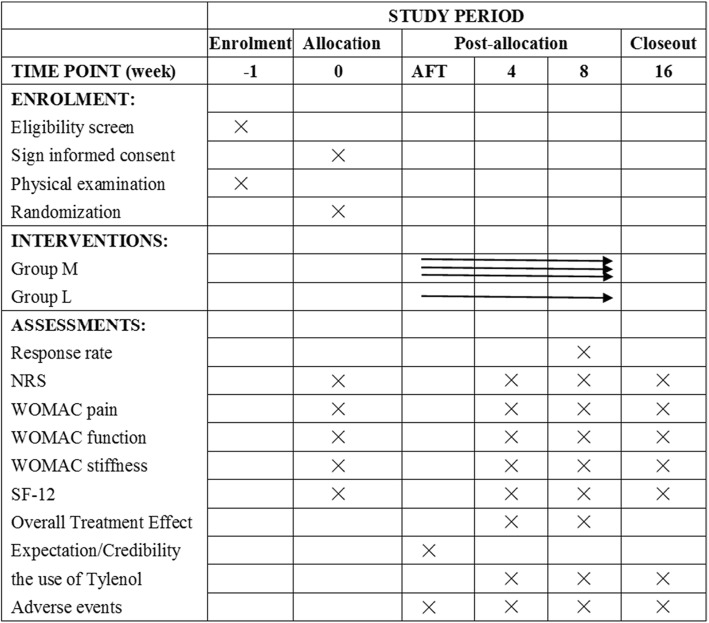
Template of content for the schedule of enrolment, interventions and assessments. AFT after first treatment; NRS numerical rating scale; SF-12 Medical Outcomes Study 12-item Short-Form Health Survey; WOMAC Western Ontario and McMasters Universities Osteoarthritis Index

This is a pragmatic, parallel, two-armed randomized controlled exploratory study with 8-week follow-up. The protocol has been registered on ClinicalTrials.gov (No NCT03359603) and will be conducted in accordance with the Declaration of Helsinki. The study will be reported in accordance with the Consolidated Standards of Reporting Trials (CONSORT) statement and its relevant extensions to randomized pilot and feasibility trials [27]. The protocol was reported in accordance with the Standard Protocol Items (SPIRIT) (Additional file 1) .The study patient flow outline is shown in Figs. 1 and 2.

### Study setting, recruitment and ethics

The recruitment will be conducted at Beijing Hospital of Traditional Chinese Medicine Affiliated to Capital Medical University. The study plans to recruit 60 patients with knee osteoarthritis using acupuncture students to do the recruiting. The study was approved by the medical ethical review committee of Beijing Hospital of Traditional Chinese Medicine Affiliated to Capital Medical University (No. 2017BL-076-01). Patients will be recruited through WeChat (a social networking site), outpatient units and print advertisements. People willing to participate in the study will be invited to contact the clinical research coordinator (CRC) by telephone. The CRC will perform a preliminary screening of people for inclusion and exclusion criteria, and then, if appropriate, schedule a face-to-face baseline visit with a senior specialist in physical and rehabilitation medicine. Patients will also be referred for radiographic evaluation. All eligible patients will provide written informed consent (Additional file 2). The CRC will discuss the study in detail (i.e., the study purpose, procedures and time commitment, as well as the potential risks and benefits associated with participation in the study) with potential patients and obtain written informed consent. The confidentiality of patient records will be protected. At the time of enrolment, each patient will be assigned a unique randomization number, which is the only direct identifier included on all case report forms.

### Inclusion criteria

1. Age 45–75 years, male or female.
2. Diagnosis of knee osteoarthritis according to the American College of Rheumatology (ACR) criteria [28].
3. Symptoms have been present for more than 6 months.
4. Radiologic confirmation of knee osteoarthritis (Kellgren–Lawrence grade [29] II or III in the recent 6 months).
5. An average knee pain severity over the past week of ≥ 4 out of 10 on an 11-point numerical rating scale (NRS) [30].
6. Agreed to refrain from the use of any analgesics during the trial (paracetamol sustained-release tablets will be given to patients if their pain intensity reaches ≥ 8 out of 10 on an 11-point NRS).
7. Signed informed consent.

### Exclusion criteria

1. History of knee surgery or waiting for surgery (knee replacement or knee arthroscopy).
2. Knee pain caused by other diseases (such as joint loose bodies, severe effusion of the joint cavity, infection, malignant tumors, autoimmune diseases and trauma).
3. History of arthroscopy within 1 year or intra-articular injection within 4 months.
4. History of receiving acupuncture treatment within 3 months.
5. Severe acute/chronic organic or mental diseases.
6. Pregnant and lactating women.
7. Coagulation disorders (such as hemophilia, etc.).
8. Participation in another clinical study in the past 3 months.
9. History of a cardiac pacemaker, metal allergy or needle phobia.

### Randomization and blinding

Patients will be randomly assigned (on a 1:1 ratio) to the more frequent acupuncture treatment group (group M) or the less frequent acupuncture treatment group (group L). The randomization sequence will be computer generated by independent research staff with SAS 9.3 software, using block randomization with a block size of six via a central randomization system. The randomization sequence will be embedded into the software (Beijing Guide Technology Co, Ltd). The CRC will input the patient information on a tablet computer and will be given a random number. The randomization protocol will be designed by a statistician (Y. Wang) of Fuwai Hospital, China Academy of Medical Science who is not involved in the later statistical work of the study.

The outcome assessor and data analyst will be blinded to group assignments. The acupuncturist and patients will not be blinded because of the nature of the intervention. Patients’ allocated interventions will not be revealed until the statistical analysis is completed.

### Interventions

The treatment strategies for acupuncture are generally considered a pragmatic compromise between the need for some standardization and the need for individualization. This treatment is based on a distinguished Chinese textbook, as well as traditional Chinese medicine meridian theory to treat *Bi* syndrome. Furthermore, the therapy was discussed with experts in the field of acupuncture.

The most important local points in the literature were chosen as obligatory points. Therefore, the following five local points have to be used for every treatment session on the affected knee: ST 35, EX-LE5 (Neixiyan), LR 8, GB 33 and an Ahshi point. Another three customized acupoints should be chosen for therapy in accordance with the individual localization of the meridians that traverse the area of the most painful point, with the intent of optimizing efficacy. ST34, ST36, ST32, ST40 and EX-LE2 will be used for pain at the stomach meridian. GB31, GB36, GB34, GB39 and GB41 will be used for pain at the gallbladder meridian. BL39, BL40, BL57 and BL60 will be used for pain at the bladder meridian. LR7, SP9, SP10, KI10, SP4, SP6, LR3 and KI3 will be used for pain at the three-yin meridians of the foot (Table 1). The selection of the three customized acupoints will be based on the experience of the acupuncturist. Therefore, the number of needles used is eight (unilateral).

**Table 1:** Locations for acupuncture

Acupoint	Location
ST 35 (Du bi)	On the anterior aspect of the knee, in the depression lateral to the patellar ligament
EX-LE5 (Nei xi yan)	On the anterior aspect of the knee, in the depression medial to the patellar ligament
LR8 (Ququan)	On the medial aspect of the knee, in the depression medial to the tendons of the semitendinosus and the semimembranosus muscles, at the medial end of the popliteal crease
GB33 (Xi yang guan)	On the lateral aspect of the knee, in the depression between the biceps femoris tendon and the iliotibial band, posterior and proximal to the lateral epicondyle of the femur
Ashi point	The point where the patient feels most pain
ST32 (Fu tu)	On the anterolateral aspect of the thigh, on the line connecting the lateral end of the base of the patella with the anterior superior iliac spine, 6 cun^a^ superior to the base of the patella
ST 34 (Liang qiu)	On the anterolateral aspect of the thigh, between the vastuslateralis muscle and the lateral border of the rectus femoris tendon, 2 cun superior to the base of the patella
ST 36 (Zu san li)	3 cun directly below ST35, and one finger-breadth lateral to the anterior border of the tibia
ST40 (Feng long)	On the anterolateral aspect of the leg, lateral border of the tibialis anterior muscle, 8 cun superior to the prominence of the lateral malleolus
EX-LE2 (He ding)	On the anterior aspect of the thigh, in the depression superior to the base of the patella
GB31 (Fengshi)	On the lateral aspect of the thigh, in the depression posterior to the iliotibial band where the tip of the middle finger rests, when standing up with the arms hanging alongside the thigh
GB 34 (Yang ling quan)	On the fibular aspect of the leg, in the depression anterior and distal to the head of the fibula
GB 36 (Waiqiu)	On the fbular aspect of the leg, anterior to the fibula, 7 cun proximal to the prominence of the lateral malleolus
GB39 (Xuanzhong)	On the fbular aspect of the leg, anterior to the fibula, 3 cun proximal to the prominence of the lateral malleolus
GB41 (Zulin qi)	On the dorsum of the foot, distal to the junction of the bases of the fourth and fifth metatarsal bones, in the depression lateral to the fifth extensor digitorum longus tendon
BL39 (Wei yang)	On the posterolateral aspect of the knee, just medial to the biceps femoris tendon in the popliteal crease
BL40 (Wei zhong)	On the posterior aspect of the knee, at the midpoint of the popliteal crease
BL57 (Cheng shan)	On the posterior aspect of the leg, at the connecting point of the calcaneal tendon with the two muscle bellies of the gastrocnemius muscle
BL60 (Kun lun)	On the posterolateral aspect of the ankle, in the depression between the prominence of the lateral malleolus and the calcaneal tendon
LR 3 (Tai chong)	In the depression anterior to the junction of first and second metatarsal bones
LR 7 (Xi guan)	On the tibial aspect of the leg, inferior to the medial condyle of the tibia, 1 cun posterior to SP9
KI 3 (Tai xi)	On the posteromedial aspect of the ankle, in the depression between the prominence of the medial malleolus and the calcaneal tendon
KI10 (Yin gu)	On the posteromedial aspect of the knee, just lateral to the semitendinosus tendon, in the popliteal crease
SP4 (Gong sun)	On the medial aspect of the foot, anteroinferior to the base of the first metatarsal bone, at the border between the red and white flesh
SP6 (San yin jiao)	On the tibial aspect of the leg, posterior to the medial border of the tibia, 3 cun superior to the prominence of the medial malleolus
SP 9 (Yin ling quan)	On the tibial aspect of the leg, in the depression between the inferior border of the medial condyle of the tibia and the medial border of the tibia
SP 10 (Xuehai)	On the anteromedial aspect of the thigh, on the bulge of the vastus medialis muscle, 2 cun superior to the medial end of the base of the patella

^a^1 cun (≈ 20 mm) is defined as the width of the interphalangeal joint of the patient’s thumb

Treatment will be performed with sterilized disposable steel needles, 0.30 mm × 25 mm or 0.30 mm × 40 mm (Huatuo disposable acupuncture needle; Suzhou, Jiangsu, China). The depth of needling should be 10–30 mm, depending on the point location. Needles will be stimulated manually for 10 s to achieve “De Qi”. Paired alligator clips will be attached transversely to the needle holders at LR 8-GB 33 and two other customized acupoints. An electric stimulator (HANS-200A acupoint nerve stimulator; Jisheng, Nanjing, China) will be applied to the four acupoints. The electro-acupuncture stimulation will last for 30 min with a dilatational wave of 2/100 Hz and a current intensity (0.1–1.0 mA) depending on the patient’s comfort level (preferably with skin around the acupoints shivering mildly without pain). Other needles will be manipulated for 10 s during the treatments. If both knees are affected, both knees will be treated with acupuncture as indicated. However, the electric stimulator will be used on only one knee (more painful).

The acupuncturist will be required to have a Chinese medicine practitioner license and have been qualified for at least 10 years. Paracetamol sustained-release tablets (Tylenol; Shanghai Johnson Pharmaceutical Co., Ltd.) will be given to patients if their pain intensity is ≥ 8 out of 10 on an 11-point NRS. Acupuncture treatment will be discontinued if the patients suffer from any adverse events and the acupuncture physicians can decide to terminate the trial.

#### Group M

Thirty patients in group M will receive acupuncture treatment of 3 sessions per week (every Monday, Wednesday and Friday) for 8 weeks. Patients will not be allowed to take any analgesic or anti-inflammatory medications during the trial. In cases of intolerable knee pain, the patients will be instructed to take Tylenol as a rescue medication. The usage of Tylenol will be documented by the outcome assessors. The use of other treatments, such as injections of any kind, moxibustion, ear acupuncture or cupping, will not be allowed.

#### Group L

Thirty patients in group L will receive acupuncture treatment once per week (every Monday, Wednesday or Friday) for 8 weeks. Other interventions will be similar with group M.

### Outcomes

If only one knee is affected, the assessment of the outcomes will relate to this knee. If the patient has two affected knees of which only one meets the ACR criterion and Kellgren–Lawrence grade II or III, only this knee will be evaluated. In the case that both knees are affected in accordance with the inclusion criteria (ACR and Kellgren–Lawrence grade II or III), the more painful knee will be randomly chosen for evaluation.

#### Primary outcome measurement

The response rate is the percentage of patients with improvement in average pain (NRS) of at least 2 units and in the Western Ontario and McMaster Universities Osteoarthritis Index [31] (WOMAC) function subscale score of at least 6 units at week 8 compared with baseline. The pain NRS is a self-administered instrument, a number selected from 0 to 10 by the patient. The intensity of pain range (over the past week) is from 0 = no pain to 10 = worst possible pain. The WOMAC function subscale refers to the participant’s ability to move around and perform usual activities of daily living. It is comprised of 17 questions (each question ranged from 0 to 4) regarding the degree of difficulty experienced due to osteoarthritis in the study of the knee, with higher scores indicating worse physical function.

#### Secondary outcome measurements

Knee pain will be assessed by the NRS and the WOMAC pain subscale (five items, scored from 0 to 20, higher scores indicating worse pain) at weeks 4, 8 and 16. The WOMAC function subscale will be used to measure physical function at weeks 4, 8 and 16. The WOMAC stiffness subscale will be used to measure the degree of stiffness experienced in the knee at weeks 4, 8 and 16. The Overall Treatment Effect will be used to measure the overall acupuncture treatment effect at weeks 4 and 8. The 12-item Short Form Health Survey [32] (SF-12; scored from 0 to 100, higher scores representing better quality of life) will be used to measure the quality of life at weeks 4, 8 and 16. The Expectation and Credibility of treatment rating scale [33] will be used to measure the patients’ attitudes to acupuncture after the first treatment. The number of adverse events and severe adverse events and the usage of Tylenol will be recorded from baseline to week 16.

#### Data collection, management and monitoring

To promote patient retention and complete follow-up, all interventions are free to patients.

The Case Report Form (CRF) will be completed on paper copies and then entered into the Excel spreadsheet by an independent investigator to act as the first level of control to ensure the accuracy of the data. The second level of data integrity will include data monitoring and validation that will be conducted on a regular basis throughout the study. The original CRFs and all other forms (including the consent forms) will be archived securely at the School of Acupuncture-Moxibustion and Tuina, Beijing University of Chinese Medicine. The Research Ethical Committee of Beijing Hospital of Traditional Chinese Medicine Affiliated to Capital Medical University will audit the trial conduct every 5 months independently of investigators and the sponsor, and will decide on any premature closure of the study.

### Sample size

The current study is designed as a pilot study to explore symptomatic improvement in patients with knee osteoarthritis receiving 3 sessions per week of acupuncture compared to 1 session per week. Acupuncture as a complex intervention is different from drugs. If the acupoints in the study were changed, then the effectiveness of the acupuncture would be altered. Consequently, we do not use data from the literature to calculate the sample size. We based our sample size on the response rate. Based on our previous study (unpublished) and clinical experience, the sample size is calculated to provide 80% power if 70% of group M achieved a decrease ≥ 2 points on the NRS and a decrease ≥ 6 points in WOMAC function score at 8 weeks, compared with 30% of group L, at a two-tailed α level of 0.05. This requires a total of 60 participants, allowing for 20% dropout.

### Statistical analysis

Analyses will be intention-to-treat (ITT), with all patients included in their assigned treatment group and receiving at least one session treatment. The level of significance will be set at 0.05 (two-sided). Patients’ baseline characteristics will be summarized by treatment arm. For continuous outcomes, the data will be presented as mean (standard deviation) or the median (interquartile range) according to the normality of the distribution. Enumeration data will be presented as a percentage. For the primary outcome, we will calculate the response rates at 8 weeks and compare group M and group L using the χ^2^ test. For secondary outcomes, continuous variables including WOMAC pain, function and stiffness scores, NRS, SF-12, the Expectation and Credibility of acupuncture treatment and Overall Treatment Effect will be compared between the two groups at all follow-up time points using an unpaired Student’s *t* test or Wilcoxon rank-sum test as appropriate. For the dropout analysis, we will use the last observation carried forward method. SPSS version 21.0 (IBM SPSS Statistics, New York, USA) will be used for statistical analysis.

### Ethics and dissemination

Ethics approval was obtained from the Research Ethical Committee of Beijing Hospital of Traditional Chinese Medicine Affiliated to Capital Medical University (No. 2017BL-076-01). This trial has also been registered on ClinicalTrials.gov (No. NCT03359603) and will be reported in compliance with the CONSORT statement as well as Standards for Reporting Interventions in Clinical Trials of Acupuncture (STRICTA) [34]. Patients are included after receiving information about the study and signing the informed consent. The results of the pilot study will be disseminated in a peer-reviewed journal.

## Discussion

This study is designed to explore an effective acupuncture method for knee osteoarthritis.

We chose a frequency of 3 sessions per week as one kind of electro-acupuncture intervention, because 3 sessions per week is most commonly used in clinical practice in China to treat chronic pain. We chose a frequency of once per week as another intervention, because electro-acupuncture treatment once per week was usually used in previous studies. The study is a single-site randomized clinical trial. A single-site trial might be helpful in the aspect that the quality of the study procedure will be better controlled in a single site than in multiple sites. One potential limitation of this study is that the unblinded treatment might theoretically influence reporting or measurement of the outcomes, thus introducing bias. Therefore, we will use an Expectation and Credibility of treatment rating scale to measure the patients’ attitudes to acupuncture.

The information obtained from the exploratory study will inform the decision to conduct a full-scale randomized controlled trial to test the frequency dependence of acupuncture, which will add to the current small body of high-quality evidence that is available on the optimal number of sessions of acupuncture for the management of knee osteoarthritis.

## Trial status

This trial is currently recruiting patients.

## Additional files

Additional file 1: Completed Standard Protocol Items: Recommendation for Interventional Trials (SPIRIT) 2013 Checklist: items addressed in this clinical trial protocol. (DOC 115 kb)

Additional file 2: Model consent form (DOCX 26 kb)

## Data Availability

The full protocol for the study will be available from the corresponding author. Datasets generated or analyzed during the current study will not be publicly available due to data privacy. Results from the trial will be published in peer-reviewed international journals and positive, negative and inconclusive results will be published.

## Abbreviations

ACR: American College of Rheumatology
CONSORT: Consolidated Standards of Reporting Trials
CRC: Clinical research coordinator
CRF: Case Report Form
ITT: Intention to treat
NRS: Numerical rating scale
STRICTA: Standards for Reporting Interventions in Clinical Trials of Acupuncture
WOMAC: Western Ontario and McMaster Universities Osteoarthritis Index
