# Supplementary material for: Acupuncture of different treatment frequency in knee osteoarthritis: a protocol for a pilot randomized clinical trial
Source: Trials. 2019 Jul 11;20:423. doi: 10.1186/s13063-019-3528-8 (PMC6625113; doi:10.1186/s13063-019-3528-8)
Supplement: Supplementary file 2 — Model consent form (DOCX 26 kb) [file 13063_2019_3528_MOESM2_ESM.docx]

**知情同意书告知页**

（版本号：1.0 日期：2017年09月25日）

我们将要开展一项“不同频次针刺治疗膝骨关节炎的随机对照临床研究”，您的情况可能符合该项研究的入组条件，因此，我们想邀请您参加该项研究。本知情同意书将向您介绍该研究的目的、步骤、获益、风险、不便或不适等，请仔细阅读后慎重做出是否参加研究的决定。当研究者向您说明和讨论知情同意书时，您可以随时提问并让他/她向您解释您不明白的地方。您可以与家人、朋友以及您的医生讨论之后再做决定。

若您目前正参加其他临床研究，请告知您的研究医生或者研究人员。

本项研究的项目负责人是首都医科大学附属北京中医医院刘存志教授，本项研究的资助方是北京市科委重大项目。

**为什么进行本项研究？**

膝骨关节炎又称退行性膝关节病、增生性膝关节炎，是临床上常见的关节疾病之一，常发生于中老年人尤其是女性。膝骨关节炎患病率约为23.9%，其高发病率与高致残率不仅给患者带来沉重经济负担，也严重影响患者生活质量。

目前对于膝骨关节炎的治疗多采取轻中度对症治疗、重度手术治疗的相关措施。药物治疗膝骨关节炎近期疗效不错，但往往随着停药时间的延长而复发，且大部分患者出现不同程度的不良反应。针灸治疗膝骨关节炎的历史悠久，安全性高、无副作用，应用广泛，受到广大患者的欢迎。但医学界目前对于是否推荐针灸治疗膝骨关节炎存在争议，急需循证医学证据进一步验证针灸治疗膝骨关节炎的疗效。

**哪些人将被邀请参加本项研究？**

本项研究将邀请符合以下纳入排除标准（以下任意一项为“否”不能参加试验。）：

①年龄45-75岁之间，男女不限；

②单/双侧膝关节疼痛，病程超过6个月；

③6个月以内的放射检查显示KL分级Ⅱ或Ⅲ级；

④疼痛数字评分法（NRS）≥4；

⑤签署知情同意书

排除标准（以下任意一项为“是”不能参加试验。）：

① 有膝关节手术史或正在等待膝关节手术（膝关节置换或膝关节镜手术）；

② 其他疾病引起的膝部疼痛（如关节游离体、关节腔严重积液、感染、恶性肿瘤、自身免疫疾病、外伤、骨折、痛风、腰骶椎疾病等）；

③ 评价关节1年之内有关节镜检查史、近4个月有关节腔注射史；

④ 近3月内接受针灸治疗；

⑤ 严重的急性或慢性器质性或精神神经类疾病；

⑥ 凝血功能障碍疾病（如血友病等）；

⑦ 具有心脏起搏器；

⑧ 备孕、妊娠期及哺乳期妇女；

⑨ 近3个月内参加其他临床研究；

本项研究将纳入60例膝骨关节炎患者，

**本研究怎样进行？**

本研究将历时16周左右。如果您自愿参加本项研究，我们希望您配合进行一下事宜：

1、纳入研究前，您的研究医生将参照纳入排除标准，询问您的相关情况，并告知您研究的相关情况，解答所有有关的疑问；

2、如果您进入试验，您将会有相同的机会进入电针组，并接受相应的治疗，第1-8周接受每周3次或1次的针刺治疗，在第4周±1天、第8周±3天和第16周±3天进行访视并进行相关量表测评，分别通过西安大略和麦克马斯特大学骨关节炎指数、疼痛数字评分法等评价针刺对患者疼痛和功能的影响，通过健康调查简表评价针刺对患者生活质量的改善，通过整体疗效评价评估针刺整体治疗效果，通过针刺不良反应评价观察针刺治疗的安全性等。

3、整个试验期间，您可在疼痛时向医生领取乙酰氨基酚作为临时口服止痛药物，并在日记卡上详细记录服药情况。请您保管好所有的包装及剩余的药物，试验结束后您的医生将这些材料回收留档。

**参加该研究对受试者日常生活的影响？**

当您决定是否参加本研究时，请仔细考虑如上所列的检查和随访对您的日常工作、家庭生活等可能的影响。考虑每次回访的时间与交通问题。若您对试验涉及的检查和步骤有任何疑问，可以向我们咨询。

研究期间需要禁止服用非本试验提供的药物。

考虑到您的安全以及为确保研究结果的有效性，在研究期间您不能再参加其他任何有关药物和医疗器械的临床研究。

**参加本研究患者有哪些风险？**

您的研究医生将监控整个研究中出现的不良反应。若在研究期间，您发生任何副作用或不适，请您即刻向研究医生报告，这是至关重要的。研究医生将全面了解您的情况，制定下一步措施，对症治疗相关不适。如果您或您的研究医生认为您无法耐受这些副作用，您可能会退出本研究。

**针刺的风险**

我们既往开展的大量针灸临床研究显示，针刺可能出现较轻的不良事件主要为皮下血肿、晕针和针后遗感。

**参加本研究可能的获益**

研究表明，针刺可以镇痛，改善膝骨关节炎相关症状。本研究应用每周3次的针刺治疗膝骨关节炎，可能能够改善膝痛、功能障碍等症状，提高患者生活质量。同时我们希望从您参与的这项研究中得到的信息在将来能够使与您病情相同的病人获益。

**如果不参加此研究，有没有其他备选治疗方案？**

您可以选择不参加本项研究，这对您获得常规治疗不会带来任何不良影响。目前针对您的健康情况，常规的治疗方法有：口服非甾体类抗炎药、止痛药、葡萄糖氨基酸、仙灵骨葆胶囊等中成药或汤药、局部外用药物、关节腔注射或关节镜手术，严重者可选择关节置换。

**是否一定要参加并完成本项研究？**

您是否参加本研究是完全**自愿的**。如果您不愿意，可以拒绝参加，这对您目前和未来的卫生医疗不会有任何负面影响。即使您同意参加本研究之后，您也可以在任何时间退出试验，你不会因推出试验而遭到歧视或报复，也不会影响您获得正常的医疗服务。当您决定不再参加本研究时，希望您及时告知您的研究医生，以便研究医生就您目前的健康状况提供建议和指导。如果您选择参加本项研究，我们希望您能够坚持完成全部研究过程。

出现以下情况我们将终止您参加研究：

- 出现严重不良事件者，根据医生判断应该停止该病例临床试验者。
- 出现其他影响试验观察的病证，根据医生判断应该停止临床试验者，作无效病例处理。
- 临床试验方案实施中发生了重要偏差，如依从性太差等，难以评价针刺疗效。
- 受试者在临床试验过程中不愿意继续进行临床试验，向主管医生提出退出临床试验的要求者。

监管机构也可能在研究期间终止本研究。如果发生本研究提前终止的情况，我们将及时通知您，您的研究医生会根据您的健康状况为您下一步的治疗计划提供建议。

对于中途退出的受试者，出于安全性考虑，我们有末次随访计划，您有权拒绝。除此之外，希望您将所有未用的止痛药归还您的研究医生。若您退出后，发现新的与您健康和权益相关的信息时，我们可能会再次与您联系。

受试者退出后，今后我们将不收集与其有关的新数据。并对如何处理之前收集的研究数据及因不良反应退出的数据做出细致说明。

**参加研究的费用**

### 本研究的24次或8次针刺治疗、对乙酰氨基酚的费用由研究单位承担。

本研究无报酬、交通费、误工费等补偿。

**发生研究相关伤害的处理？**

当您的健康状况在参加本研究期间受到伤害时，请告知研究者（林璐璐大夫，联系电话15628933890），我们会采取必要的医疗措施。根据我国相关法规条例规定，发生研究相关的伤害时，本项研究的课题组将承担相应的医疗费用及对此提供相应的经济补偿。

**若参加研究，我需要做什么？**

- 提供准确的既往病史和当前的病情
- 告诉研究医生您在研究期间出现的任何健康问题。
- 除试验发放的止痛药外，未经研究医生允许，不应进行任何治疗。
- 按研究医生要求接受治疗和服用止痛药，并做相应记录。
- 按要求访视，请将所有药物包装和未使用的止痛药归还给研究医生。
- 止痛药室温、密封、防潮保存，放在儿童接触不到的地方，不要将研究药物给任何人。
- 研究期间不要参加其他医学研究。
- 保持生活方式的恒定，不宜增加运动量，尽量避免剧烈运动或爬山等加重膝关节负担的运动。

**个人信息会得到保密吗？**

如果您决定参加本项研究，您参加研究及在研究中的个人资料均属保密。可以识别您身份的信息将不会透露给研究小组以外的成员，除非获得您的许可。所有的研究成员都被要求对您的身份保密。您的档案将保存在有锁的档案柜中，仅供研究人员查阅。为确保研究按照规定进行，必要时，政府管理部门或伦理委员会的成员按规定可以在研究单位查阅您的个人资料。这项研究结果发表时，将不会披露您个人的任何资料。伦理委员会将公正此项研究是安全和合乎道德的，并在赫尔辛基宣言指导下进行。

**与研究相关的新信息？**

在试验过程中我们可能会获知有关治疗的新的信息，我们会及时通知您，让您决定是否继续参加研究或退出。

**研究结束之后是否继续提供研究药物治疗？**

研究结束后，课题组将不再继续向您提供针刺和止痛药。您的医生将与您讨论以后的治疗方案。

**如果有问题或困难，该与谁联系？**

如果您有与本研究相关的任何问题，请联系林璐璐，联系电话15628933890。

如果您有与受试者自身权益相关的问题，可与首都医科大学附属北京中医医院医学伦理委员会联系，联系电话：010-64011516。

**第二部分 知情同意签名页**

**受试者知情同意声明**

我已被告知针刺治疗膝骨关节炎的随机对照临床研究的背景、目的、步骤、风险及获益情况。我有足够的时间和机会进行提问，问题的答复我很满意。我也被告知，当我有问题，或想进一步获得信息，应当与谁联系。我已经阅读这份知情同意书，并且同意参加本研究。我知道在研究期间任何时刻无需任何理由我都可以退出本研究。我被告知我将得到这份知情同意书的副本，上面包含我和研究者的签名。

患者签名：_______________ 日期：_______年___月___日

联系电话：_______________

法定代理人签字【如适用】：___________ 日期：_______年___月___日

与受试者关系：_______________

我确认，在知情同意书中的信息是被正确解释了的并且受试者和/或受试者合法代表明白理解了这些信息。受试者自愿同意参加本研究。

公平见证人签字【如适用】：___________ 日期：_______年___月___日

**研究者告知声明**

我已告知该受试者针刺治疗膝骨关节炎的随机对照临床研究的背景、目的、步骤、风险及获益情况，给予他/她足够的时间阅读知情同意书、与他人讨论，并解答了其有关研究的问题；我已告知该受试者当遇到问题时的联系方式；我已告知该受试者可以在研究期间的任何时候无需任何理由退出本研究。

研究者签名：______________ 日期：_______年___月___日

联系电话：______________ 研究单位：__________________
